# Contrasting effects of the alkaloid ricinine on the capacity of *Anopheles gambiae* and *Anopheles coluzzii* to transmit *Plasmodium falciparum*

**DOI:** 10.1186/s13071-021-04992-z

**Published:** 2021-09-15

**Authors:** Domonbabele F. D. S. Hien, Prisca S. L. Paré, Amanda Cooper, Benjamin K. Koama, Edwige Guissou, Koudraogo B. Yaméogo, Rakiswendé S. Yerbanga, Iain W. Farrell, Jean B. Ouédraogo, Olivier Gnankiné, Rickard Ignell, Anna Cohuet, Roch K. Dabiré, Philip C. Stevenson, Thierry Lefèvre

**Affiliations:** 1 Institut de Recherche en Sciences de La Santé (IRSS), Bobo Dioulasso, Burkina Faso; 2 Laboratoire Mixte International Sur Les Vecteurs (LAMIVECT), Bobo Dioulasso, Burkina Faso; 3 MIVEGEC, Université de Montpellier, IRD, CNRS, Montpellier, France; 4 Université Joseph KI-ZERBO, Ougadougou, Burkina Faso; 5 Royal Botanic Gardens, Kew, Surrey, Richmond, TW9 3AE UK; 6 Institut Des Sciences Et Techniques, Université Nazi Boni, Bobo-Dioulasso, Burkina Faso; 7 Department of Plant Protection Biology, Unit of Chemical Ecology, Disease Vector Group, Swedish University of Agricultural Sciences, Uppsala, Sweden; 8 Natural Resources Institute, University of Greenwich, Kent, ME4 4TB UK; 9 Centre de Recherche en Écologie Et Évolution de La Santé (CREES), Montpellier, France

**Keywords:** *Plasmodium falciparum*, *Anopheles coluzzii*, *Anopheles gambiae*, Ricinine, Malaria transmission, Transmission-blocking strategies

## Abstract

**Background:**

Besides feeding on blood, females of the malaria vector *Anopheles gambiae* sensu lato readily feed on natural sources of plant sugars. The impact of toxic secondary phytochemicals contained in plant-derived sugars on mosquito physiology and the development of *Plasmodium* parasites remains elusive. The focus of this study was to explore the influence of the alkaloid ricinine, found in the nectar of the castor bean *Ricinus communis*, on the ability of mosquitoes to transmit *Plasmodium falciparum*.

**Methods:**

Females of *Anopheles gambiae* and its sibling species *Anopheles coluzzii* were exposed to ricinine through sugar feeding assays to assess the effect of this phytochemical on mosquito survival, level of *P. falciparum* infection and growth rate of the parasite.

**Results:**

Ricinine induced a significant reduction in the longevity of both *Anopheles* species. Ricinine caused acceleration in the parasite growth rate with an earlier invasion of the salivary glands in both species. At a concentration of 0.04 g l^−1^ in *An. coluzzii*, ricinine had no effect on mosquito infection, while 0.08 g l^−1^ ricinine-5% glucose solution induced a 14% increase in *An. gambiae* infection rate.

**Conclusions:**

Overall, our findings reveal that consumption of certain nectar phytochemicals can have unexpected and contrasting effects on key phenotypic traits that govern the intensity of malaria transmission. Further studies will be required before concluding on the putative role of ricinine as a novel control agent, including the development of ricinine-based toxic and transmission-blocking sugar baits. Testing other secondary phytochemicals in plant nectar will provide a broader understanding of the impact which plants can have on the transmission of vector-borne diseases.

**Graphical abstract:**

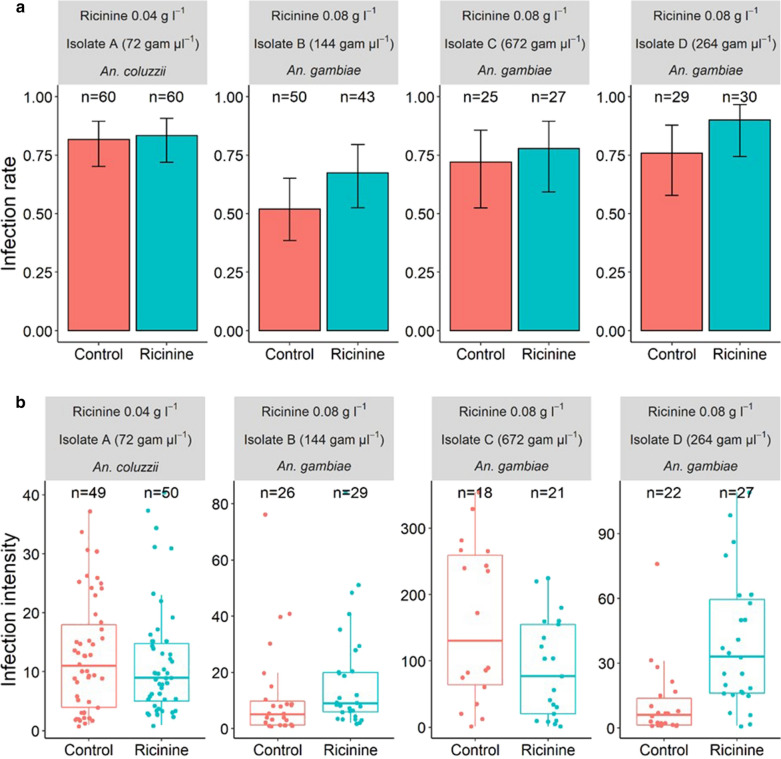

**Supplementary Information:**

The online version contains supplementary material available at 10.1186/s13071-021-04992-z.

## Background

Human malaria is a global disease caused by parasites of the genus *Plasmodium*, which are transmitted by *Anopheles* mosquitoes. Despite substantial control progress in the last 2 decades, malaria remains a major cause of morbidity and infant mortality, with an estimated 229 million cases and 409,000 deaths in 2019, mostly in sub-Saharan Africa [1]. Improving existing and available tools, such as impregnated bed nets, is essential in the immediate future; however, there is also an urgent need for the implementation of alternative solutions to achieve long-term control [2]. One approach is to disrupt the parasite development in mosquito vectors following blood-feeding on a vaccinated or drug-treated patient, i.e. transmission-blocking drugs or vaccines (TBD/V) [3].

The administration of TBD/V is not intended to protect or clear infected people, but to prevent the development of the parasite within the mosquito vector. When ingested through a blood meal, the drugs or antibodies inhibit parasite development, thereby reducing or blocking malaria transmission from the mosquito to a human host [4]. Besides blood-feeding, the delivery of a transmission-blocking agent into a mosquito host can occur through mosquito sugar-feeding and/or contact [5–7]. Regarding the latter delivery route, Paton et al. [8] showed that *Anopheles* exposure to atovaquone through treated surfaces before or shortly after *Plasmodium* *falciparum* infection can result in a full parasite arrest in the mosquito midgut.

Although female mosquitoes are well-known blood-feeders, sugar, obtained from floral and extra-floral nectaries, fruits and honeydew and phloem sap, is an important part of their diet and significantly affects the life history and transmission potential of malaria vectors [9–11]. From 1949 to 1968, Terzian et al. [12] took advantage of mosquito sugar feeding to test the sporontocidal activity of a number of synthetic antimalarial drugs on the bird malaria parasite *Plasmodium gallinaceum* in the mosquito *Aedes aegypti*. Further studies later confirmed that antimalarial drugs, such as cycloguanil or pyrimethamine, have significant sporontocidal activity against *P. falciparum* in freshly infected mosquitoes following imbibement of the test compounds mixed in a sucrose solution [13]. In contrast to administration via the blood meal, which allows enzymatic transformation by the vertebrate host, this method allows for the direct analysis of the effects of the primary drug or its known metabolites. Another major advantage of this approach is that it does not require extensive drug safety assessment as part of substantial clinical trials in humans. Besides synthetic antimalarial drugs, attention could be given to phytochemicals with similar properties.

Some plant nectars contain secondary metabolites that can limit insect infection by pathogens [14, 15]. Considering the potential of natural plant extracts to block malaria transmission during mosquito blood-feeding [16, 17], toxic secondary metabolites from plants, including nectar compounds, may similarly affect the development of malaria parasites within the mosquitoes. For instance, *Anopheles coluzzii* females that fed on the flowering ornamental plant *Thevetia neriifolia* were less likely to carry sporozoites after an infectious meal compared to females that had fed on fruits of *Lannea microcarpa* or the ornamental plant *Barleria lupulina* [18]. A better understanding of the effects of plants on mosquito vector-malaria parasites interactions can therefore potentially give rise to novel control strategies, such as the development of transmission-blocking sugar baits [5, 6].

Besides possible prophylactic or therapeutic properties against pathogens within mosquitoes, another possible benefit of plant secondary metabolites could be their adverse effects on other key transmission traits, such as mosquito lifespan. Extracts of the castor bean (*Ricinus communis*, Euphorbiaceae) contain the alkaloid ricinine, which is present in *R. communis* nectar, and have strong insecticidal [19, 20] and antimicrobial [21–23] activities, including a newly reported effect against *Plasmodium* *berghei* in mice. Thus, extracts of *R. communis* are a promising candidate for the development of transmission-blocking drugs delivered through mosquito sugar meals.

The current study aimed at evaluating the effect of ricinine on the development of *P. falciparum* in two major malaria vectors, namely *Anopheles* *gambiae* and *An.* *coluzzii*. Mosquito females were challenged with sympatric field isolates of *P. falciparum* (five distinct isolates in total) using direct membrane feeding assays, and through a series of experiments, the effects of ricinine on (i) mosquito competence for *P. falciparum*, (ii) the timing of oocyst rupture and sporozoite dissemination and (iii) female survival were examined.

## Methods

### Mosquito strains

Laboratory-reared *An. gambiae* and *An. coluzzii* were obtained from outbred colonies established in 2012 that have been repeatedly replenished with F1 from wild-caught females collected in Soumousso (11°23′14′′N, 4°24′42′′W) and the Kou Valley (11°24′N, 4°24′59′′W) and identified by SINE PCR [23]. Mosquitoes were held in 30  ×  30  ×  30-cm mesh-covered cages under standard insectary conditions (12 h:12 h light:dark [L:D], 27  ±  2 °C, 70  ±  5% relative humidity). Females were maintained on rabbit blood by direct feeding (protocol approved by the national committee of Burkina Faso; IRB registration #00004738 and FWA 00007038) and adult males and females fed with a 5% glucose solution. Larvae were reared at a density of about 300 first-instar larvae in 700 ml of water in plastic trays and fed with Tetramin Baby Fish Food (Tetrawerke, Melle, Germany).

### Ricinine detection in nectar

Naturally occurring levels of ricinine in *R. communis* nectar were estimated by collecting samples from seven plants cultivated at the Royal Botanic Gardens, Kew, and from three plants from the wild populations of *R. communis* around Bobo-Dioulasso, Burkina Faso. Plants from Kew were sampled during June and July 2016, and plants from Bobo-Dioulasso were sampled during August 2018. Extracts were analysed by liquid chromatography (LC)-electrospray ionization mass spectroscopy (ESIMS) and UV spectroscopy using a Thermo Fisher Velos Pro LC-MS. Samples (5 μl) were injected directly into a Phenomenex Luna C18 (2) column (150 Å–3 mm i.d., 3 μm particle size at 400 μl min^−1^ maintained at 30 °C and eluted using a linear gradient of 90:0:10 (*t*  =  0 min) to 0:90:10 (*t*  =  20–25 min), returning to 90:0:10 (*t*  =  27–30 min) with water:methanol:1% formic acid in acetonitrile, respectively. Compounds were detected on a Thermo Fisher Velos Pro Dual-Pressure Linear Ion Trap Mass Spectrometer. A peak corresponding to ricinine eluted after 5.60 min with *m*/*z*  =  165 [M  +  H]^+^ and 329 [2 M  +  H]^+^, consistent with a ricinine standard having a molecular formula of C_8_H_8_N_2_O_2_.

Ricinine was quantified using the LC-MS method above against an authentic ricinine standard obtained in our laboratories as described below. The mean concentration of ricinine in nectar was determined to be approximately 40 ppm (0.04 g l^−1^), with the maximum observed concentration exceeding 100 ppm. To test the effect of ricinine in mosquito diet on survival and parasite development, a medium (40 ppm) and a high concentration (80 ppm) of ricinine were incorporated into sugar feeds. The medium concentration (40 ppm) was considered ecologically relevant as it has previously been used in studies of female *An. gambiae* survival after feeding on ricinine in sugar solutions [19, 20].

### Ricinine isolation

Ricinine for the experiments described in the current study was isolated from seeds of *R. communis*, which have high concentrations of ricinine [24]. Using a protocol for ricinine extraction established at Kew, seeds (200 g) were split open, ground using a mortar and pestle and then boiled in 1 l of distilled water. The extract was then vacuum filtered with a coarse filter paper, which separated the seed remains from the liquid. The filtrate was re-filtered with a finer grade filter paper (Whatman No. 1) and then extracted with chloroform (100 ml) in a separating funnel and shaken for 1 min. This was left to separate for 1–2 h. The chloroform was then run off and another portion of chloroform (100 ml) added, shaken and left to separate. This process was repeated two additional times. After the last portion of chloroform was run off, the remaining aqueous layer was centrifuged for at least 20 min at 2000 rpm for further separation of chloroform and the aqueous layer. The combined chloroform extracts were evaporated to dryness under vacuum on a Buchi Rotovap (Buchi UK Ltd., Newmarket, UK) and recrystallised from distilled water. Crystals were collected by vacuum filtration and left to dry in a desiccator. The compound identity was confirmed by HPLC-HRMS (experimental [+] *m*/*z*  =  165.0652, calculated for C8 H9 O2 N2+  = 165.0658; uv: λmax 255, 306 nm) and by comparison of the ^1^H and ^13^C NMR spectrum acquired in MeOH-d4 at 30 °C recorded on a Bruker Avance 400=MHz spectrometer using standard pulse sequences and parameters, according to Souza et al. [25].

### Preparation of the ricinine solutions and oral administration to mosquitoes

Two distinct ricinine solutions were prepared by mixing 1 l of a 5% glucose solution with either 0.04 g or 0.08 g of ricinine powder. These two solutions, at concentrations of 0.04 g l^−1^ and 0.08 g l^−1^, were solubilised in 5 ml of pure DMSO (DMSO, Honeywell, Germany). DMSO was also added to the 5% glucose control solution. The ricinine and control solutions were kept at 4 °C. Upon emergence, batches of female mosquitoes were randomly offered the treatment for 2 to 3 consecutive days through cotton pads soaked in either the 5% glucose control solution or alternatively the 0.04 g l^−1^ or the 0.08 g l^−1^ of ricinine and 5% glucose solution and placed on top of the mosquito cages. The cotton pads were changed daily. On day 3, the cotton pads were removed from the cages and mosquitoes deprived of food for 24 h prior to the infection. Given the daily frequency of mosquito sugar feeding at 27 °C (i.e. about 75% [26]) by 2–3 days, all mosquitoes would have acquired their sugar meal treatment at least once. Male and female mosquitoes were kept together to ensure insemination. On day 4, females were transferred to 500-ml paper cups at a density of 80 mosquitoes for infection.

### Parasite isolates and mosquito experimental infection

Female mosquitoes (*An. gambiae* or *An.* *coluzzii*, depending on availability, see below) were fed with blood drawn from naturally *P. falciparum* gametocyte-infected patients recruited among 5–12-year-old school children in villages surrounding Bobo-Dioulasso, Burkina Faso, using Direct membrane feeding assays (DMFA) as previously described [18, 27]. Briefly, thick blood smears were taken from each volunteer, air-dried, Giemsa-stained and examined by microscopy for the presence of *P. falciparum* at the IRSS laboratory in Bobo-Dioulasso. Asexual trophozoite parasite stages were counted against 200 leucocytes, while infectious gametocyte stages were counted against 1000 leukocytes. Children with asexual parasitaemia of  >  1000 parasites per microlitre (estimated based on an average of 8000 leucocytes ml^−1^) were treated in accordance with national guidelines. Asymptomatic *P. falciparum* gametocyte-positive children were recruited for the study. Blood from gametocyte isolates was collected by venipuncture in heparinized tubes. Five distinct parasite isolates (named hereafter A, B, C, D and E), with respective gametocytaemia of 72, 144, 672, 264 and 88 gametocytes µl^−1^ of blood, were used for the experimental infections. DMFA was performed using whole donor blood (i.e. no serum replacement) [18]. Three-to-four-day-old female mosquitoes held in the paper cups (see above) were allowed to feed on this blood for 1 h. Non-fed or partially fed females were removed and discarded, while the remaining fully engorged mosquitoes were kept in a biosafety room under the same standard conditions (12 h:12 h L: D, 27  ±  2 °C, 70 ± 5% relative humidity). After infection, fully engorged mosquitoes were returned to 30  ×  30  ×  30-cm mesh-covered cages and provided their assigned treatment (i.e. 5% glucose or ricinine solution). Mosquito females thus received their treatment both before and after the infection.

In contrast to standard membrane feeding assays whereby mosquitoes are fed with cultured parasites, DMFA relies on naturally *P. falciparum* gametocyte-infected patients. When associated with sympatric mosquitoes, DMFA can be a reasonable approximation of what occurs in natural conditions. This requires the simultaneous availability of local mosquitoes and patients with relatively high density of gametocytes to reach sound infection rates in the mosquitoes. The parasitological surveys in the human population and the identification of gametocyte carriers can be challenging. When the opportunity of relatively high gametocytaemia arises, colony-derived mosquitoes might not always be available. In the experiments described in the current study, the availabilities of both *An. gambiae* and *An.* *coluzzii* were not simultaneously synchronized with that of the collected natural parasite isolates. In particular, the concentration of 0.04 g l^−1^ of ricinine and *An.* *coluzzii* was used for the experimental infections using parasite isolates A and E, while concentrations of 0.08 g l^−1^ and *An.* *gambiae* were used for other infections (isolates B–D). This means that ricinine concentration and mosquito species were here confounded.

### Ricinine ingestion by mosquitoes

To confirm that ricinine was ingested by the mosquitoes used in each experiment (see below), a subset of mosquitoes fed on the sugar with the 0.04 g l^−1^ ricinine regime was analysed. Fifteen mosquitoes from blood-fed parasite-positive, 10 blood-fed parasite-negative and 20 blood-unfed mosquitoes were maintained on 0.04 g l^−1^ ricinine  +  5% glucose solution and analysed. Samples consisted of five mosquitoes that were ground into 100 ml of methanol (Methanol, Fisher, Chemical) using a ball bearing to extract mosquito chemistry. These mosquito extracts (a total of nine samples: three samples of blood-fed parasite-positive, two blood-fed parasite-negative and four blood-unfed) were then analysed for the detection of ricinine, using ESIMS-LCMS, as described above.

### Experiment 1: effects of ricinine on *An. gambiae* and *An. coluzzii* susceptibility to *P. falciparum*

The susceptibility of *An. gambiae* and *An. coluzzii* to *P. falciparum* was assessed in terms of both infection rate (i.e. the proportion of female mosquitoes harbouring at least one oocyst in their midgut after the infectious blood meal) and intensity (i.e. the number of oocysts per infected mosquito). To this end, a total of four parasite isolates (*n*  =  4: isolates A–D) was used for experimental infections. On day 7/8 post-blood meal (dpbm), 160 ricinine-fed females (*n*  =  60, 43, 27 and 30 for isolates A–D, respectively) and 164 control females (those fed on 5% glucose, *n*  =  60, 50, 25 and 29 for isolates A–D, respectively) were dissected and stained with 1% mercurochrome to microscopically (× 400) assess the presence and number of oocysts, the immature, non-transmissible stage of the malaria parasite. Isolate A and a concentration of 0.04 g l^−1^ of ricinine were used to infect *An. coluzzii* and isolates B–D and a concentration of 0.08 g l^−1^ of ricinine were used to infect *An. gambiae*.

### Experiment 2: effects of ricinine on *P. falciparum* oocyst rupture in mosquito midguts and sporozoite dissemination in head/thoraces

On 9–12 dpbm, a total of 170 *An. coluzzii* (85 fed on 0.04 g l^−1^ ricinine and 85 fed on 5% glucose and using parasite isolate A), 109 *An. gambiae* (54 fed on 0.08 g l^−1^ ricinine and 55 fed on 5% glucose and using isolate C) and 108 other *An. gambiae* (49 fed on 0.08 g l^−1^ ricinine and 59 fed on 5% glucose using isolate D) females were dissected daily (range: 10–31 females per day and isolate) to microscopically assess the presence and number of oocysts in mosquito midguts (isolate A) and qPCR detection of sporozoites in head/thoraces (isolates A, C, D). *Anopheles gambiae* females infected with isolate B were not used because they were all dissected as part of experiment 1 (see above).

Oocyst rupture in the mosquito midgut and sporozoite invasion of salivary glands are highly asynchronous. While some oocysts are intact and keep developing on 9–12 dpbm, others have already ruptured and released their sporozoites [26, 27]. To explore possible differences in the timing of sporozoite dissemination in mosquito salivary glands between ricinine-fed and control females, three metrics were measured [26, 27]:

(i) The proportion of infected mosquitoes with ruptured oocysts on 10–12 dpbm; the number of mosquitoes with at least one ruptured oocyst in their midguts out of the total number of infected mosquitoes (i.e. harbouring either intact and/or ruptured oocysts).
(ii) The proportion of ruptured oocysts on 10–12 dpbm; for each infected mosquito, the number of ruptured oocysts out of the total number of oocysts (intact  +  ruptured).
(iii) The proportion of oocyst-infected mosquitoes with disseminated sporozoites in their head and thorax on 10–12 dpbm; the number of oocyst-infected mosquitoes harbouring sporozoites in their head/thoraces on 10–12 dpbm out of the total number of infected mosquitoes (i.e. harbouring either intact and/or ruptured oocysts).

### Experiment 3: effects of ricinine on *An. gambiae* and *An. coluzzii* survival

To determine how parasite infection and ricinine interact to influence mosquito longevity, three membrane feeding assays were performed following the same general procedure as described above except that a group of uninfected control mosquitoes were added. Uninfected control mosquitoes received heat-treated gametocytic blood (isolates C–E) to kill parasite gametocytes, as previously described [28]. For each group (ricinine-fed and exposed to gametocytic blood, ricinine-fed and exposed to heat-treated gametocytic blood, control and exposed to gametocytic blood, and control and exposed to heat-treated gametocytic blood), between 20 and 42 females were placed in 15  ×  15  ×  15-cm cages. Dead mosquitoes were removed and counted in each cage (12 cages, i.e. 4 cages for each isolate C–E) every morning at 08:00. Dead mosquitoes exposed to infectious blood were individually stored in 1.5-ml tubes (Eppendorf, Hamburg, Germany) at − 20 °C to determine their infection status using qPCR (see below). Isolates C, D and a concentration 0.08 g l^−1^ of ricinine were used to infect *An. gambiae*, and isolate E and a concentration of 0.04 g l^−1^ of ricinine were used to infect *An. coluzzii.*

### *Plasmodium falciparum* DNA extraction and qPCR

*Plasmodiumfalciparum* genomic DNA was extracted from mosquito head-thorax by mechanical grinding tissues using Tissulyser II in an extraction buffer (0.1 M Tris HCl, pH 8.0, 0.01 M EDTA, 1.4 M NaCl, 2% cetylltrimethyl ammonium bromide) [29]. The grinding obtained was incubated at 65 °C for 10 min. Total DNA was extracted with chloroform, precipitated in isopropanol, washed in 70% ethanol and resuspended in sterile water. Parasite detection was carried out by qPCR, using *P. falciparum* mitochondrial DNA-specific primers 5′-TTACATCAGGAATGTTTTGC-3′ and qPCR-PfR 5′-ATATTGGGATCTCCTGCAAAT-3′ [30].

### Statistical analyses

All statistical analyses were performed in R (version 3.6.3) [31]. Experiment 1: logistic regression by generalized linear models (GLM) was used to test the effect of ricinine 0.04 g l^−1^ on the prevalence of oocysts in *An. coluzzii* (isolate A). We also used a binomial GLM to test the effect of ricinine 0.08 g l^−1^, isolates (B–D) and their interaction on the prevalence of oocysts in *An. gambiae*. A GLM with negative binomial errors was used to test the effect of ricinine 0.04 g l^−1^ on the oocyst intensity (isolate A). A negative binomial GLM was also used to test the effect of ricinine 0.08 g l^−1^, isolates (B–D) and their interaction on the oocyst intensity. Experiment 2: logistic regression by linear models was used to test the effect of ricinine on (i) the proportion of infected mosquitoes with ruptured oocysts (isolate A, 0.04 g l^−1^ ricinine) (binomial GLM), (ii) the fraction of ruptured oocysts (isolate A, 0.04 g l^−1^ ricinine) (quasibinomial GLM because of overdispersion) and (iii) the proportion of oocyst-infected mosquitoes with sporozoites in their head and thorax (isolates A, C and D) (binomial GLM). Experiment 3: the effect of ricinine, infection and interaction on mosquito survivorship was analysed using Cox’s proportional hazard regression model. Model simplification used stepwise removal of terms, followed by likelihood ratio tests (LRT). Term removals that significantly reduced the explanatory power (*P * <  0.05) were retained in the minimal adequate model [32]. R scripts and raw datasets are described in Additional files 1, 2, 3, 4.

## Results

### Ingestion of ricinine by mosquitoes

Mosquitoes experimentally administered ricinine from all three feeding groups (blood-fed parasite positive, blood-fed parasite negative and blood-unfed) were found to have a detectable amount of ricinine using ESIMS-LCMS analysis. Of the nine samples analysed, two of the three blood-fed parasite-positive samples, both of the blood-fed parasite-negative samples and all of the four blood-unfed samples had detectable levels of ricinine, ranging from 1 to 10 µM.

### Experiment 1: effects of ricinine on *An. gambiae* and *An. coluzzii* susceptibility to *P. falciparum*

**Fig. 1:**
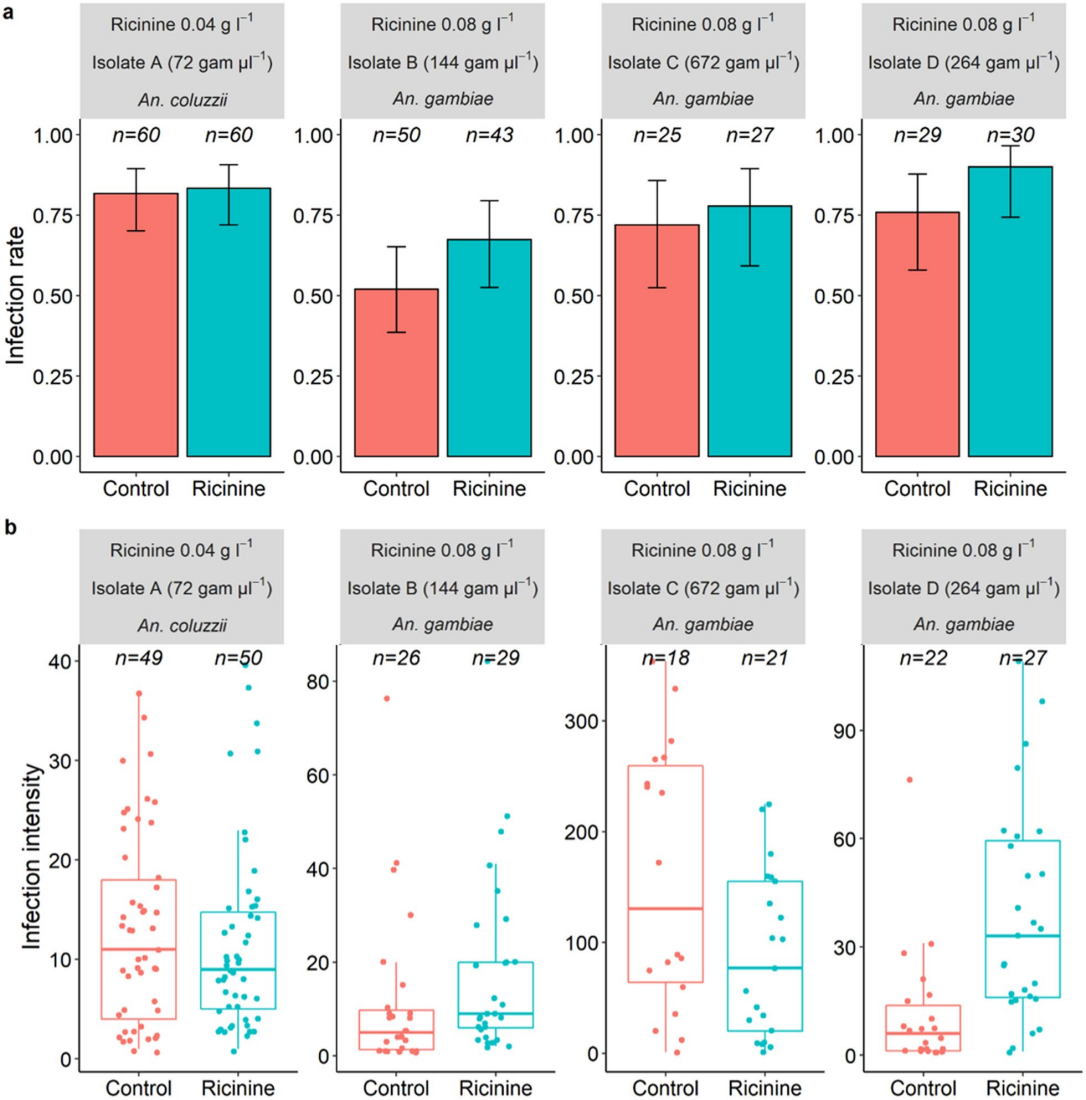
Effect of ricinine on the susceptibility of *Anopheles gambiae* and *An. coluzzii* to four natural isolates of *Plasmodium falciparum* (A, B, C and D). **a** Infection rate (± 95% CI) on day 7 post-blood meal (dpbm), expressed as the number of females harbouring at least one oocyst in their midguts out of the total number of dissected females for each treatment (red bars: control mosquitoes fed with a 5% glucose solution; blue bars: test mosquitoes fed with a 5% glucose solution and either 0.04 or 0.08 g l^−1^ of ricinine) and for each of four parasite isolates (A–D). **b** Infection intensity at 7 dpbm, expressed as the number of developing oocysts in midguts of infected females, for each treatment and the four parasite isolates. A concentration of 0.04 g l^−1^ of ricinine and *An. coluzzii* were used for the first experimental infection using parasite isolate A (left panel in **a** and **b**), while concentrations of 0.08 g l^−1^ and *An. gambiae* were used for other infections (isolates B, C and D). “g µl^−1^” corresponds to the number of gametocytes per microlitre of blood

At a concentration of 0.04 g l^−1^, ricinine had no effect on *An.* *coluzzii* infection rate (LRT *X*^2^  =  0.06, *df*  =  1, *P*  =  0.81, Fig. 1a, left panel) or intensity (LRT *X*^2^  =  0.5, *df * =  1, *P* =  0.48, Fig. 1b, left panel). However, at a concentration of 0.08 g l^−1^, ricinine increased the overall infection rate of *An. gambiae* by 14% (parasite isolates B–D, Fig. 1a, LRT *X*^2^ = 4.5, *df * =  1, *P* = 0.03). This increase was consistent across all three parasite isolates used to infect the mosquitoes, i.e. no isolate by treatment interaction was observed (LRT *X*^2^  =  0.58, *df*  =  2, *P* =  0.75). The effect of the higher concentration of ricinine (0.08 g l^−1^) on infection intensity, however, differed across parasite isolates, i.e. a significant isolate by treatment interaction was observed (LRT *X*^2^  =  16.51, *df* =  2, *P* =  0.0003, Fig. 1b). While 0.08 g l^−1^ ricinine increased the number of developing parasites in mosquitoes exposed to isolates B and D, mosquitoes exposed to isolate C tended to have a decreased number of parasites (Fig. 1b).

### Experiment 2: effect of ricinine on *P. falciparum* oocyst rupture in mosquito midguts and sporozoite dissemination in heads/thoraces

**Fig. 2:**
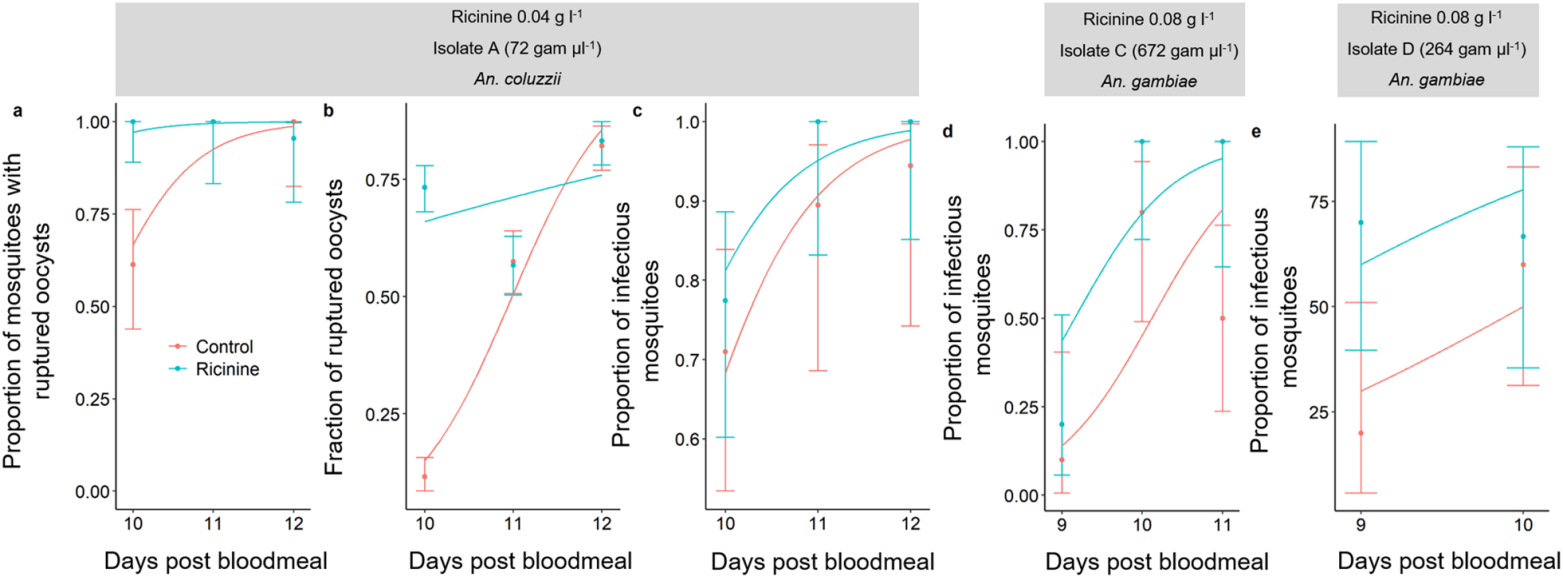
Effect of ricinine on *Plasmodium falciparum* oocyst rupture in mosquito midguts and sporozoite dissemination in head/thoraces for three parasite isolates (A, C and D). **a** Proportion of infected mosquitoes with ruptured oocysts (± 95% CI) from 10 to 12 dpbm, expressed as the number of mosquitoes with at least one ruptured oocyst out of the total number of infected mosquitoes, i.e. harbouring either intact and/or ruptured oocysts, in response to the ricinine treatment (blue) or the control (red). The lines represent best-fit logistic growth curves for each treatment. **b** Fraction of ruptured oocysts (± 95% CI), expressed as the number of ruptured oocysts out of the total number of oocysts (intact  +  ruptured). The lines represent best-fit logistic growth curves for each isolate. **c** Proportion of mosquitoes with disseminated sporozoites in the head/thorax (± 95% CI). Sample size =  7–31 individuals/dpbm/isolate/treatment (mean  =  14.75). A concentration of 0.04 g l^−1^ of ricinine and *Anopheles coluzzii* was used for the first experimental infection using parasite isolate A (panels **a**–**c**), while concentrations of 0.08 g l^−1^ and *An.* *gambiae* were used for other infections (isolates C and D)

A total of 140 *An. coluzzii* females (72 fed on 0.04 g l^−1^ of ricinine and 68 on control diet) exposed to parasite isolate A were dissected daily from 9 to 12 dpbm (between 18 and 31 females/day/treatment) to microscopically assess the presence and number of oocysts (intact and ruptured) in the mosquito midguts and for the qPCR detection of sporozoites in head/thorax. Ricinine increased the proportion of mosquitoes with ruptured oocysts (LRT *X*^2^  =  12.8, *df*  = 1, *P* =  0.0003, Fig. 2a): all mosquitoes (100%) from the ricinine treatment exhibited at least one ruptured oocyst in their midgut by 10 dpbm compared to 61% in the control group (Fig. 2a). In addition, the fraction of ruptured oocysts in mosquito midguts was higher in the ricinine treatment than in the control (LRT *X*^2^  =  109, *df*  =  1, *P* < 0.0001, Fig. 2b). Finally, the proportion of mosquitoes with disseminated sporozoites in their head/thorax, the most epidemiologically relevant metric, was higher in ricinine-fed mosquitoes compared to controls from 9 to 12 dpbm (LRT *X*^2^  =  18.9, *df*  = 1, *P* <  0.0001, Fig. 1c–e), regardless of the parasite isolate, i.e. no treatment by isolate interaction (LRT *X*^2^  =  1.2, *df* =  2, *P* =  0.55). Moreover, sporozoite dissemination in the head/thorax of ricinine-fed mosquitoes occurred earlier than in control (significant dpbm by treatment interaction: LRT *X*^2^ = 4.36, *df* = 1, *P* =  0.037, Fig. 2c, d). Together, these results suggest that ricinine increased the maturation of *P. falciparum*.

### Experiment 3: effects of ricinine on *An. gambiae* and *An. coluzzii* survival

**Fig. 3:**
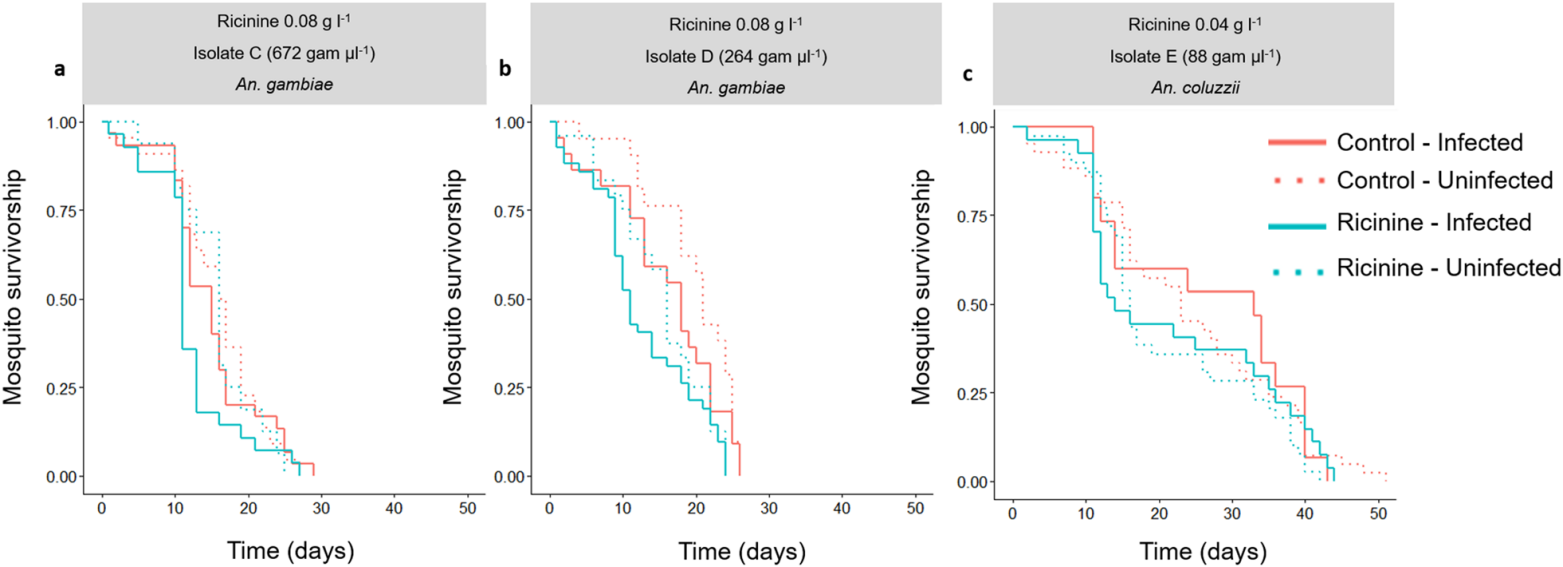
Effect of ricinine on the longevity of uninfected and *Plasmodium falciparum*-infected *Anopheles* *gambiae* and *An. coluzzii* using three parasite isolates (C, D and E). A concentration of 0.08 g l^−1^ of ricinine was used to test its effect on the survival of *An. gambiae* exposed to isolates C and D (**a**, **b**), while a concentration of 0.04 g l^−1^ was used to test its effect on the longevity of *An. coluzzii* exposed to isolate E (**c**). Mosquitoes were monitored until all individuals died. Sample size  =  16–42 individuals/treatment/infection/isolate (mean  =  34)

The survival of (i) ricinine-fed mosquitoes exposed to *P. falciparum* (*n* =  106); (ii) ricinine fed and unexposed, i.e. received a heat-treated gametocytic blood meal (*n* =  97), mosquitoes; (iii) glucose-fed control mosquitoes and females exposed to *P. falciparum* (*n* =  103) and (iv) glucose-fed control and unexposed (*n* =  86) mosquitoes were monitored from 1 to 50 dpbm, when the last mosquito died. The DNA of *P. falciparum*-exposed dead mosquitoes was extracted to detect the presence of *P. falciparum* using qPCR. Mosquitoes (ricinine-fed or glucose-fed control), which remained uninfected upon parasite exposure, were excluded from the analysis to focus on the effect of infection and ricinine (0.04 g l^−1^ and 0.08 g l^−1^) on mosquito survival. Ricinine exerted a toxic effect, reducing mosquito median survival by 3.5 days (*n* =  152 5% glucose control females and 176 ricinine-fed females, LRT *X*^2^ =  10.7, *df* = 1, *P* = 0.001, hazard ratio =  1.35; 95% CI  =  1.09–1.69, Fig. 3). This was true regardless of the mosquito infection status (interaction between infection and treatment: LRT *X*^2^ = 0.005, *df* =  2, *P* =  0.94, Fig. 3) and parasite isolate (interaction between isolate and treatment: LRT *X*^2^ =  0.7, *df* =  2, *P* = 0.7, Fig. 3). Mosquito survival was not influenced by infection (LRT *X*^2^ = 0.64, *df* =  1, *P* =  0.42) and there was no infection by isolate interaction (LRT *X*^2^  =  4.5, *df * =  2, *P* =  0.1). *An. coluzzii* used for isolate E lived longer than *An. gambiae* used for isolates C and D (LRT *X*^2^ =  65.6, *df*  =  2, *P* < 0.001). Finally, there was no three-way interaction among treatment, infection and isolate (LRT *X*^2^ = 0.88, *df* =  2, *P* =  0.64).

## Discussion

In this study, laboratory assays were conducted to evaluate the impact of the alkaloid ricinine on the (i) survival and (ii) susceptibility of female *An.* *gambiae* and *An. coluzzii* to natural isolates of *P.* *falciparum* as well as (iii) the parasite growth rate. Overall, our results demonstrated that the consumption of ricinine in glucose solution decreased the lifespan of both *An. gambiae* and *An. coluzzii*, increased *An. gambiae* susceptibility to infection at a concentration of 0.08 g l^−1^ and accelerated parasite development in both mosquito species.

Although secondary metabolites contained in plant nectars function primarily as a defence against herbivores, there can be a variety of potential benefits and costs for nectar feeders to acquire these phytochemicals [33]. In particular, secondary compounds, including phenolic acids and flavonols, may enhance insect longevity [34], plant attractiveness to pollinators [35] or protection against pathogens [36, 37]. However, the role of alkaloid secondary compounds in insect-plant interactions remains elusive. Some alkaloids can be beneficial, e.g. enhance honeybee memory formation through odour cues associated with food rewards [38]. However, consumption of alkaloids is generally costly in that they reduce insect survival and fecundity [39]. Consistent with previous studies on *Anopheles* mosquitoes [19, 20], we found that mosquitoes fed with 0.04 g l^−1^ or 0.08 g l^−1^ of the alkaloid ricinine and 5% glucose solution displayed reduced survival compared to controls. Nyasembe et al. [20] observed that 50% of *An. gambiae* fed a 0.04 g l^−1^ ricinine in a 6% glucose solution were dead within 4 days, while here we report a median survival time of 12 days in ricinine-fed *An. gambiae* and *An. coluzzii*. A possible explanation for the observed discrepancy could be the status of insecticide resistance in the mosquito colonies used in this study. The outbred *An. gambiae* and *An. coluzzii* colonies are derived from females collected recently in the villages of Soumousso and Bama, respectively, where the level of phenotypic insecticide resistance is found to be highly associated to elevated activity of enzymes of detoxification [40]. A high level of metabolic resistance to insecticides could concomitantly be associated with an enhanced ability to detoxify plant secondary metabolites [41]. Future studies exploring the effect of ricinine and other plant secondary compounds on the survival of insecticide resistant vs. susceptible individuals will be required to confirm this possibility in mosquitoes.

The transmission potential of *Plasmodium* parasites by *Anopheles* vectors is extremely sensitive to variation in mosquito survival [41, 42]. Small changes in mosquito lifespan can result in relatively large changes in transmission potential [41, 42]. As such, ricinine delivered through attractive sugar baits in field settings could result in decreased malaria transmission. On the other hand, our data indicate that oocyst rupturing and sporozoite invasion of mosquito salivary glands occur earlier in ricinine-fed mosquitoes, suggesting that ricinine actually facilitates growth acceleration of *P. falciparum* within the mosquito gut. The transmission of *P. falciparum* is also sensitive to variation in the extrinsic incubation period (EIP) of the parasite, in which a shortened EIP inevitably results in an earlier development of infection in the mosquitoes and thereby a higher risk of transmission. While the mechanism underlying the ricinine-enhanced developmental rate of *P. falciparum* is unknown, ricinine may exert a direct effect on the *Plasmodium* parasites, through stimulation of DNA replication and/or ATP production. The shortened EIP and decreased mosquito lifespan induced by ricinine may have a contrasting effect on malaria transmission. These results would need to be combined into an epidemiological model to predict the contribution of ricinine to overall malaria transmission potential.

Shorter EIP in ricinine-fed mosquitoes could illustrate a case of adaptive phenotypic plasticity in response to decreased mosquito survival, which reduces future opportunities of transmission. However, it is currently unclear whether malaria parasites can accelerate their sporogonic cycle when their transmission is compromised by the imminent death of their mosquito vector [44]. Such condition-dependent developmental strategies, described in blood-stage malaria parasites [44, 45], deserve consideration in infected mosquitoes.

Anti-parasite effects of nectar alkaloids have been demonstrated in bumblebees [14, 46]. In particular, the consumption of the alkaloids anabasine, nicotine and gelsemine significantly reduced the load of *Crithidia bombi*, a protozoan parasite of bumblebees by 81, 61 and 100%, respectively [14, 46]. In the present study, at a concentration of 0.04 g l^−1^, ricinine had no effect on *An. coluzzii* infection. At a concentration of 0.08 g l^−1^, our findings indicate a 14% increase in *An. gambiae* infection rate. Similarly, ricinine tended to increase the infection load of *An. gambiae*, although this effect was inconsistent across the parasite isolates used. The precise mechanisms behind this effect are not yet clear but interactions among mosquito detoxification of ricinine, body condition and immune responses can be suspected. In particular, *Anopheles* mosquitoes could be subject to a trade-off between immune response and survival such that when they are maintained on a ricinine-glucose solution, they allocate more energy to detoxification, to retrieve lifespan perspective, at the cost of immune response. If this hypothesis is correct, we would also expect fecundity reduction in ricinine-fed mosquitoes.

Eight of the nine mosquito samples analysed had detectable levels of ricinine, ranging from 1 to 10 µM. This indicates that ricinine was ingested concurrently with sugar feeding, which is consistent with the observation of Nyasembe et al. [20], who detected ricinine in mosquito midguts after feeding on dosed sugar solutions. Future studies are required to quantify the level of sugar intake by control mosquitoes and ricinine-fed mosquitoes. Because of its bitter taste, it is possible that sugar intake (amount and frequency) in treated mosquitoes was reduced and resulted in undernourished, poorly vigorous mosquitoes. This could also explain the increased infection level and decreased longevity observed in these mosquitoes.

Examples of an increase in infection following the ingestion of a toxic molecule by an insect are scarce in the literature. The results reported here are comparable to those obtained by Terzian et al. [48] wherein the oocyst density of *P.* *gallinaceum* varied in a dose-dependent manner in the gut of *Ae.* *aegypti* maintained on sulfadiazine- or PABA-4% sugar solutions. This relationship was characterized by a low-dose stimulation of PABA or sulfadiazine and a high-dose inhibition resulting in an inverted U-shaped dose response, similar to hormesis. Repeating experiment 1 using a higher dose of ricinine would allow confirming this possibility. To maximize the success of mosquito feeding success, females were here starved of sugar solution for 24 h before receiving the infectious blood meal. During this 24-h period it is possible that ricinine concentration in the mosquito tissues dropped to a level ineffective on the early phases of parasite development. Future experiments, suppressing or reducing the mosquito starvation period, would be required to possibly reach higher concentrations of ricinine in the guts when the early and most fragile stages of the parasite begin the sporogonic cycle.

## Conclusions

Overall, our findings reveal that the consumption of the nectar alkaloid ricinine can have unsuspected and contrasting effects on key phenotypic traits that govern the intensity of malaria transmission. Future work is needed before concluding on the possible role of ricinine as a novel control agent, including the development of ricinine-based toxic and transmission-blocking sugar baits. Testing other secondary phytochemicals in plant nectar will provide a broader understanding of plant-mediated effects on the transmission of vector-borne diseases.

## Supplementary Information

**Additional file 1.** Data for experiment 1 used for Fig.1 and statistical analyses.

**Additional file 2.** Data for experiment 2 used for Fig.2 and statistical analyses.

**Additional file 3.** Data for experiment 3 used for Fig.3 and statistical analyses.

**Additional file 4.** Rscript used for all Figures and statistical analyses.

## Data Availability

All data and R code files are included within the article’s additional files.

## Abbreviations

dpbm: Days post-blood meal
L:D: Light dark
qPCR: Real-time polymerase chain reaction
DMFA: Direct membrane feeding assay
